# Loss of Histone Methyltransferase KMT2D Attenuates Angiogenesis in the Ischemic Heart by Inhibiting the Transcriptional Activation of VEGF-A

**DOI:** 10.1007/s12265-023-10373-x

**Published:** 2023-03-22

**Authors:** Xiang-Min Meng, Shu-Bao Liu, Tian Deng, De-Yong Li, Lu You, Hao Hong, Qi-Pu Feng, Bing-Mei Zhu

**Affiliations:** 1 Regenerative Medicine Research Center, West China Hospital, Sichuan University, Chengdu, Sichuan China; 2 Animal Experiment Center, West China Hospital, Sichuan University, Chengdu, Sichuan China

**Keywords:** Myocardial ischemia, Angiogenesis, Endothelial cell, Histone methyltransferase, Histone methylation modification

## Abstract

Angiogenesis occurred after myocardial infarction (MI) protects heart failure (HF). The aim of our study was to explore function of histone methyltransferase KMT2D (MLL4, mixed-lineage leukemia 4) in angiogenesis post-MI. Western blotting showed that KMT2D protein expression was elevated in MI mouse myocardial. Cardiomyocyte-specific *Kmt2d*-knockout (*Kmt2d*-cKO) mice were generated, and echocardiography and immunofluorescence staining detected significantly attenuated cardiac function and insufficient angiogenesis following MI in *Kmt2d*-cKO mice. Cross-talk assay suggested that Kmt2d-KO H9c2-derived conditioned medium attenuates EA.hy926 EC function. ELISA further identified that VEGF-A released from Kmt2d-KO H9c2 was significantly reduced. CUT&Tag and RT-qPCR revealed that KMT2D deficiency reduced *Vegf-a* mRNA expression and enrichment of H3K4me1 on the *Vegf-a* promoter. Moreover, KMT2D silencing in ECs also suppressed endothelial function. Our study indicates that KMT2D depletion in both cardiomyocytes and ECs attenuates angiogenesis and that loss of KMT2D exacerbates heart failure after MI in mice.

**Supplementary Information:**

The online version contains supplementary material available at 10.1007/s12265-023-10373-x.

## Introduction

Coronary heart disease (CHD)-induced heart failure remains the leading cause of morbidity and mortality. Sudden blockage of coronary arteries or extreme reduction of blood flow can lead to MI injury and excessive apoptosis of cardiomyocytes. Angiogenesis and arteriogenesis, which are activated by ischemia and hypoxia following MI, can protect the adverse ventricular remodeling and heart failure by restoring blood supply and oxygenation in the ischemic myocardium [1]. Briefly, hypoxia-inducible factor 1 α (HIF-1α) expression is induced by hypoxia in local myocardial tissue during MI, and HIF-1α further activates downstream target genes encoding *VEGF-A* and other angiogenic growth factors to promote angiogenesis [2, 3]. Recent studies have shown that improving the EC function, promoting the formation of collateral circulation, and increasing the capillary density in the ischemic region have been suggested as novel strategies for treating myocardial ischemia [4, 5].

Recently, histone modifications and the expression of histone modifying enzymes are associated with myocardial hypertrophy, myocardial ischemia/reperfusion injury, and heart failure[6, 7]. One of the most important epigenetic regulatory machineries, histone methylation, mainly occurs on the side chains of lysine or arginine and is regulated by histone methyltransferases (HMTs) and histone demethylases (KDMs) [8]. As a common and complex type of protein posttranslational modifications (PTMs) of histones, histone methylation can regulate transcriptional activation or suppression of genes and thus participate in the occurrence and progression of many critical diseases including heart disease and cancer [9–11].

KMT2D is a member of the mammalian histone H3 lysine 4 (H3K4) methyltransferase family. Canonically, KMT2D is an essential regulator of the promoter regions or enhancer regions to induce H3K4me1 or H3K4me2 modification, which is involved in regulating chromatin accessibility and transcriptional activation of target genes [12].Previous studies have shown that KMT2D is essential for heart development [13]. We recently demonstrated the critical function of KMT2D in the adult MI mouse model [14]. Our present observations show impaired angiogenesis in the ischemic mouse heart when KMT2D was deleted specifically in the cardiomyocytes. However, the function of KMT2D in angiogenesis is not yet completely understood. Therefore, we designed this study to investigate whether and how KMT2D is involved in the regulation of angiogenesis following MI.

Angiogenesis and microvascular network reconstruction in ischemic myocardium remains a major challenge in promoting cardiac regeneration and protecting the further MI injury. The normal cellular composition of the adult heart includes cardiomyocytes, endothelial cells, smooth muscle cells, fibroblasts, and other cell populations [15]. Meanwhile, there is an extensive intercellular communication network between cardiomyocytes and non-cardiomyocytes, and the cellular cross-talk is the key to maintain cardiac homeostasis, cardiac regeneration, and angiogenesis after myocardial injury [15, 16]. In the present study, we focused on the endogenous angiogenetic response upon the myocardial ischemia and identified the regulatory effect of KMT2D on angiogenesis in the cardiomyocytes and the vascular endothelial cells by using KMT2D cardiomyocyte-specific knockout mice and cell culture system with CRISPR/Cas9 gene-edited or siRNA-mediated *KMT2D* knockdown cells.

## Methods

### Mice

The experimental protocol was approved by the committee on the Ethics of Animal Experiments of West China Hospital of Sichuan University (no. 20220216006). All efforts were made to minimize suffering in animals. At the end of the experiment, mice were anesthetized with 2% isoflurane and euthanized by cervical dislocation.

Male C57BL/6 J mice aged 8–10 weeks were purchased from GemPharmatech Co.Ltd., (Chengdu, China) and raised at the specific pathogen-free laboratory animal facility of West China Hospital, Sichuan University (Chengdu, China). *Kmt2d* floxed (*Kmt2d *^*flox/flox*^) mice were crossed with *Tnnt2-rtTA;TetO-Cre* transgenic mice (*Tnnt2*-rtTA activated *TetO*-Cre expression takes place in cardiomyocytes following doxycycline treatment) to generate *Kmt2d *^*flox/flox*^; *Tnnt2*-Cre mice, a conditional and inducible *Kmt2d* knockout mice. 10-week-old *Kmt2d *^*flox/flox*^; *Tnnt2*-Cre and their littermates *Kmt2d *^*flox/flox*^ control mice were treated with 2 mg/ml doxycycline in drinking water containing 5% sucrose for 2 weeks to induced the cardiomyocyte-specific knockout (cKO) of the *Kmt2d* gene. All mice were maintained under a 12-h light/dark cycle at 22 °C before initiation of the experiments. Randomized grouping was used, and the same group of mice were co-housed with less than 5 animals per cage.

### MI Surgery

Left anterior descending coronary artery (LAD) was ligated to create the MI mice model, as we applied before [14]. All mice were anesthetized in the chamber at 2% isoflurane, under sterile conditions, LAD was tied by a 7–0 silk suture. Sham-operated mice were treated with the same surgery without tying the left anterior descending coronary artery. Electrocardiography was performed to evaluate the successful generation of the MI model. After that, mice were sacrificed at various time points, and myocardial tissue was collected for further analysis.

### Electrocardiogram

Electrocardiogram (ECG) was performed immediately after MI surgery. All mice were anesthetized with isoflurane, carefully positioned on the ECG recording platform. Surface lead II ECG was obtained. After electrode setup and system adjustment were accomplished, the next 1 min of ECG recordings were analyzed by LabChart 8.2.3 (AD Instruments, Australia).

### Immunofluorescent Staining for Cardiac Tissues and ECs

Myocardial tissue was cryo-embedded by optical coherence tomography, and then, 10-μm cryo-sections were generated. Cryo-sections and glass bottom plates (cultured endothelial cells) were fixed with 4% paraformaldehyde, permeabilized with 0.5% Triton X-100, and blocked with 2% bovine serum albumin and incubated with different primary antibodies at 4 °C overnight according to the corresponding experiments, including Anti-Cardiac Troponin I, CD31, VEGF-A, α-SMA, wheat germ agglutinin (WGA) (L4895, Sigma-Aldrich, 1: 100 dilution), and KMT2D. The next day, Alexa-488-(SA00013-2, Proteintech, 1: 1000) or Alexa-568-conjugated secondary antibodies (A11031, Invitrogen, 1: 1000) were incubated for 1 h, and finally, the nuclei were stained with DAPI (D9542, Sigma). All sections and glass bottom plates were examined by confocal microscope (ECLIPSE Ti A1, Nikon).

### TUNEL Staining

Cryo-sections were fixed with 4% paraformaldehyde, permeabilized with 0.5% Triton X-100, and blocked with 2% bovine serum albumin and incubated with Anti-Cardiac Troponin I at 4 °C overnight. The next day, the TUNEL assay was performed using the TUNEL BrightRed Apoptosis Detection Kit (Vazyme, China) according to the manufacturer’s instructions. DAPI was used for nuclear staining, and the number of TUNEL-positive nuclei was counted. All sections were examined by confocal microscope (ECLIPSE Ti A1, Nikon).

### Western Blotting

Myocardial tissue and cell suspension were lysed by RIPA lysis buffer (MB-030–0050, Multi Sciences Biotech). BCA Protein Assay Kit (23225, ThermoFisher) was adopted to determine protein concentrations. After equal quantities, total proteins were separated in SDS-PAGE gel (4–20%, ACE Biotechnology), and the bands were transferred onto PVDF membranes (03010040001, Merck). After being blocked with 5% skimmed milk and washed with tris-buffered saline containing 0.1% tween-20 (TBST), protein bands were blotted with primary antibodies at 4 °C overnight (antibody information is provided in Supplementary Table 3). After washing with TBST, protein bands were blotted with HRP-conjugated secondary antibody and monitored using ECL buffer (32209, ThermoFisher). Quantifications of Western blotting were measured with Fusioncapt advance software.

### Echocardiography

All mice underwent transthoracic echocardiography under 1.5% isoflurane anesthesia. The cardiac structure (left ventricular internal diastolic/systolic diameter and LVIDd/LVIDs) and end-diastolic volume (EDV), end-systolic volume (ESV), and cardiac function (left ventricular ejection fraction, LV EF, and fractional shortening, FS) were detected by Vivid7 Dimension system (GE, USA) with a 30-MHz central frequency scan head and measured from M-mode images taken from the parasternal short-axis view at papillary muscle level.

### Histological Analysis

Mouse myocardial tissue was fixed in 4% paraformaldehyde followed by embedded with paraffin. Pieces of the heart embedded in paraffin were cut into 5-μm sections and mounted on glass slides. After dewaxing and dehydrating with xylene and ethanol, the sections were stained successively with hematoxylin and eosin staining, iron hematoxylin staining, Ponceau acid magenta, phosphomolybdate staining, and aniline blue dyeing. Images were acquired using a bright field microscope (Olympus, Japan). Myocardial infarct scar area and LV wall thickness were analyzed by ImageJ.

### CRISPR/Cas9 Gene Editing

The generation of KMT2D knockout H9c2 cardiomyocytes cell lines based on CRISPR/Cas9 gene-editing knockout system has been reported in our previous study [14]. Briefly, the oligodeoxynucleotides of gRNAs were annealed and cloned into PX458 plasmids (48138, Addgene), named PX458-*Kmt2d*-gRNA1 and PX458-*Kmt2d*-gRNA2, respectively, and confirmed by sequencing. H9c2 cells were detached by 0.25% trypsin–EDTA solution (25200–056, Gibco) and transfected with CRISPR/Cas9 PX458-*Kmt2d* gRNA1/2 or empty control plasmids by Nucleofector™ X Kit (V4XC-2024, Lonza) according to the manufacture’s protocol, then plated in DMEM with 10% FBS without pen/strep. Cells were cultured for 48 h at 37 °C. The PX458 plasmid contained a green fluorescent protein (GFP) marker that can be used to screen GFP-positive cells for *Kmt2d*-KO monoclonal H9c2 cell line by aseptic flow cytometry.

### siRNA and Transfection

EA.hy926 ECs were plated in a 6-well plates. After growing to 50% confluence, transfection was done with Lipofectamine 3000 according to the manufacturer’s instructions. Each siRNA was used at 20-nM final concentrations, and Nc siRNA was used as the control group. The efficiency of siRNA was detected by at 48 h after siRNA transfection. The sequences of *KMT2D* siRNAs are shown in Supplementary Table 1.

### Cardiomyocyte-Conditioned Medium (CM)

WT and *Kmt2d-*KO H9c2 cardiomyocytes were cultured in 10-cm plates. After growing to 60–70% confluence, the medium was replaced with 5% FBS DMEM. After that, the cells were incubated under normoxia or hypoxia (1% O_2_) conditions for 24 h for collection of H9c2-CM. The CM was centrifuged at 3000 rpm for 5 min at 4 °C and stored at −80 °C.

### CCK-8 Assay

ECs were seeded in 96-well plates. After growing to 50–60% confluence, the medium was replaced with H9c2 cardiomyocytes-derived CM. After further incubation for 24 h, the medium was replaced with DMEM containing 10% CCK8 reaction solution. After further incubation with CCK-8 reagent for 1 h, cell viability was determined by measuring absorbance at 450 nm.

### Cell Wound Healing Assay

ECs were seeded in 6-well plates and cultured to reach 90% confluence. ECs were treated with a proliferation blocker mitomycin C (10 μg/ml) for 2 h prior to the wound healing. A straight line in the cell monolayer was generated by scratching with a sterile 200 μl pipette tip across the culture plate. Floating cells were removed, and ECs were cultured in DMEM for 24 h. For the cross-talk assay, ECs were incubated with H9c2 cardiomyocytes-CM from different sources. After that, scratches were photographed using microscope and the wound closure was measured and quantified with ImageJ software.

### Cell Migration Assay

After 8-h incubation with serum-free DMEM, the ECs were collected through 0.25% trypsin digestion, washed, counted, and resuspended in the serum-free DMEM, 100 μl (5 × 10^4^) cell suspension was added to the upper chamber of the Transwell, and 650 μl DMEM containing 10% FBS was added to the 24-well plates. After 24 h of incubation under normoxia or hypoxia conditions, ECs were fixed in 4% paraformaldehyde and stained with 0.1% crystal violet. The number of migrated cells were photographed under microscope. For the cross-talk assay, 24-well plates were added with CM from different sources and incubated for 48 h. The rest of the experiment follows the same procedure.

### Tubule Formation Assay

ECs were resuspended in the serum-free DMEM, and then, 200 μl (8 × 10^4^) cell suspension was added to a 48-well plate precoated with Matrigel (356,234, BD). After 6–8 h of incubation under normoxia or hypoxia conditions, the tubule formation was visualized by microscope and the master junction, branch length, and number of meshes were quantified with ImageJ software. For the cross-talk assay, ECs were incubated with CM from different sources for 24 h and then seeded in 48-well plates precoated with Matrigel. The rest of the experiment follows the same procedure.

### Enzyme-Linked Immunosorbent Assay (ELISA)

ELISA was performed to measure the levels of angiogenesis-related growth factors in CM with or without hypoxia preconditioning in WT H9c2 and *Kmt2d*-KO H9c2 cardiomyocytes. Quantitative measurement of angiotensin II (Ang II), VEGF-A, endothelin-1 (ET-1), and transforming growth factor beta 1 (TGF-β1) was performed in CM supernatants using Ang II ELISA Kit (CEA005Ra, Cloud-Clone Corp), VEGF-A ELISA Kit (EK383/2, MultiSciences), ET-1 ELISA Kit (CEA482Ra, Cloud-Clone Corp), and TGF-β1 ELISA Kit (EK981, MultiSciences), respectively, according to manufacturer’s manuals.

### Cleavage Under Targets and Tagmentation (CUT&Tag)

CUT&Tag was applied to detect the DNA fragments modified by histone methylation in WT H9c2 and *Kmt2d*-KO H9c2 cardiomyocytes, the Hyperactive Universal CUT&Tag Assay kit (TD903, Vazyme) was used in accordance with the manufacturer’s instructions. Briefly, 8 × 10^4^ cells from each group were collected and incubated with 10-μl ConA beads for 10 min. After incubation, the mixture was placed on a magnetic separator and the supernatants were removed. The mixture was resuspended with 50-μl antibody buffer, after which 1 μl of H3K4me1 antibody (ab176877, Abcam) was added to each sample and mixed thoroughly, and all samples were incubated overnight at 4 °C. The mixture was placed on a magnetic separator and the supernatants were removed, and goat anti-rabbit IgG (HA1012 HUABIO, 1: 100) diluted with 50-μl Dig-wash buffer was added to the mixture for further incubation for 1 h. After that, the supernatant was removed and the mixture was washed gently with Dig-wash buffer, and 100 μl pA/G-Tnp (diluted with Dig-300 buffer, 1: 50) was added to each sample. After 1 h incubation, all samples were washed gently with Dig-300 buffer. 50 μl TTBL was added to the mixture, after which all samples were incubated in a PCR Amplifier for 1 h at 37 °C. Next, 5 μl proteinase K, 100 μl buffer L/B, and 20 μl DNA extract beads were added to the fragmented samples, vortexed and mixed thoroughly, and incubated at 55 °C for 10 min. The mixture was placed on a magnetic separator and the supernatants were removed, after which the mixture was washed gently with buffer WA and buffer WB. All buffers were removed, and the samples were allowed to air dry at room temperature for 5 min. After that, 22-μl ultrapure water was added to the centrifuge tube and the DNA was thoroughly eluted. After 5 min, the mixture was placed on a magnetic separator and 20 μl of supernatant was collected and stored at − 20 ℃.

### RT-qPCR

Total RNA was extracted from tissues or cells with a FastPure Cell/Tissue Total RNA Isolation Kit (RC101-01, Vazyme). After cDNA synthesis using HiScript Q RT SuperMix Kit (R122-01, Vazyme), RT-qPCR was conducted by qPCR using ChamQ Universal SYBR qPCR Master Mix (Q711-02, Vazyme) on ABI QuantStudio6 Q6 Real-time PCR system (ABI, USA). The relative expression of mRNA was calculated by ΔΔCt method according to standard methods. The primer sequences used for RT-qPCR analyses are listed in Supplementary Table 2.

### Statistical Analysis

Data from the mouse and cell model were expressed as mean ± SD. Significant differences were assessed either by Independent-sample *t* test (two-tailed) was used for statistical comparisons between 2 groups, or one-way ANOVA followed by Tukey’s post hoc test was used for statistical comparisons between multiple groups. A value of *P* < 0.05 was considered to be statistically different. Analyses were performed using GraphPad Prism 8.

## Results

### Dynamic Evaluation of Angiogenesis After MI

**Fig. 1:**
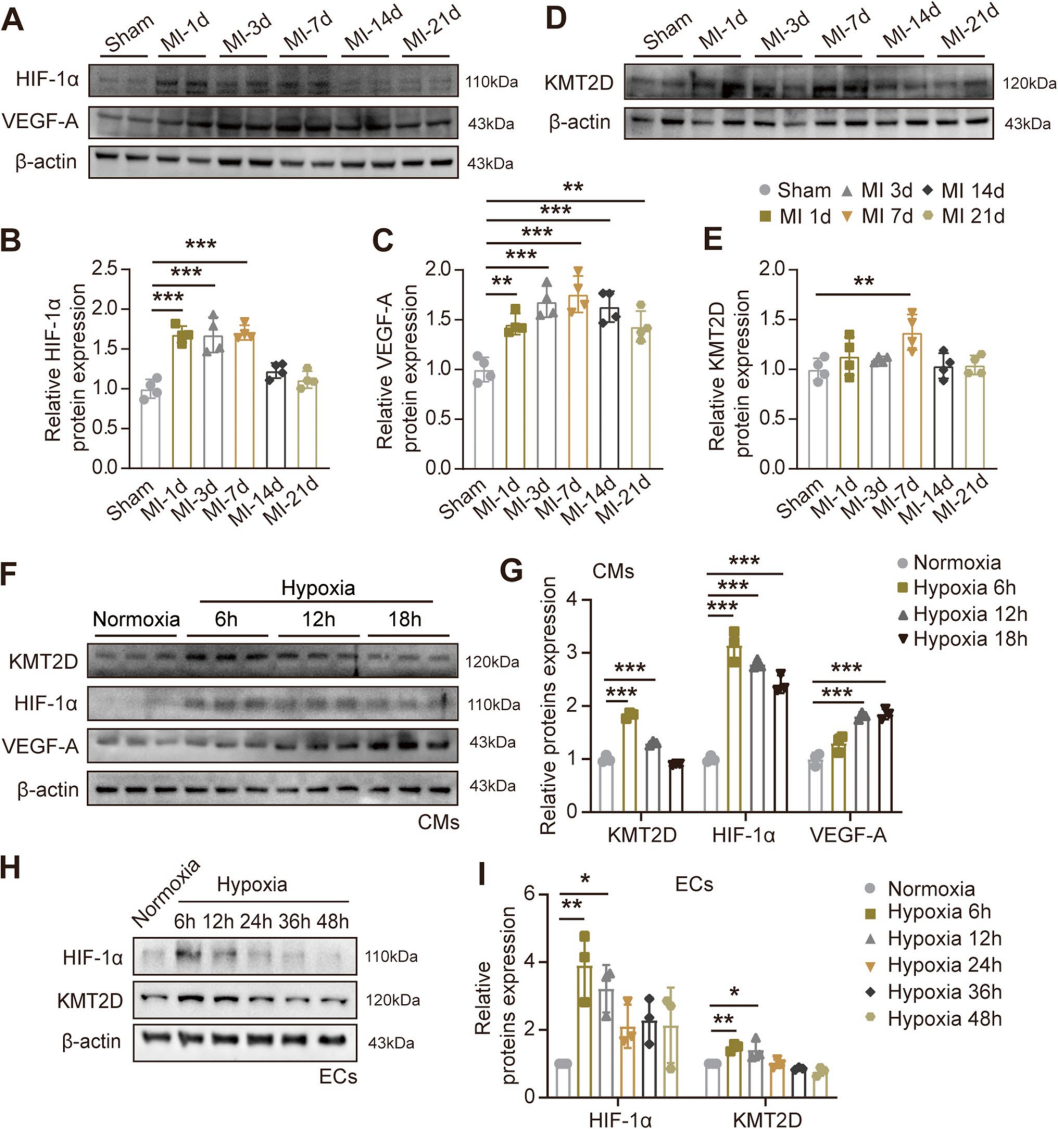
KMT2D shows a dynamic expression pattern in ischemic myocardial tissue and hypoxic cells. **A**, **D** Representative images of western blotting and **B**, **C**, and **E** quantification analysis of proteins expression in mouse heart tissues of border area from different time points after MI (*n* = 4). **F** Representative images of Western blotting and (**G**) quantification analysis of proteins expression in H9c2 cardiomyocytes at different time points after hypoxia exposure (*n* = 3). **H** Representative images of Western blotting and **I** quantification analysis of proteins expression in EA.hy926 ECs at different time points after hypoxia exposure (*n* = 3). Data are shown as mean ± SD. One-way ANOVA followed by Tukey’s post hoc test was used for statistical comparisons between multiple groups. ^*^P < 0.05, ^**^P < 0.01, and.^***^P < 0.001

We initially generated mouse myocardial infarction model via the ligation of the LAD (Figure S1A) to explore the vascular angiogenic response of the heart after different stages of myocardial infarction. Successful generation of the acute MI model was verified by electrocardiography monitoring with the substantial ST segment elevation (Figure S1B). Local myocardial tissue hypoxia during MI is associated with the induced protein expression of HIF-1α, and the upregulated HIF-1α protein expression was confirmed by Western blotting in border zone myocardium from day 1 to day 7 after MI, and compared with sham myocardium (Figs. 1A and B). Meanwhile, the crucial growth factor VEGF-A was increased 1.45-fold as early as day 1 and reached the expression peak at day 7 (Fig. 1A, C). Subsequently, the immunofluorescence staining and quantification for the myocardium sections taken from the border zone showed that CD31-positive endothelial capillaries signal was expanded at day 1 post-MI and peaked at day 3 (Figure S1C, D). With the aggravation of myocardial injury and apoptosis, CD31 fluorescent signal dropped gradually from day 3 to day 21 (Figure S1C, D). Moreover, consistent with capillary density, the numbers of α-SMA positive arterioles around the border zone increased significantly at days 3, with a peak at 3–7 days (Figure S1E, F). These results suggest that the formation of collateral vessels in border zone is present to provide inflow after MI. These results indicate that day 3 to day 7 post-MI in mice is the critical period of angiogenesis.

### KMT2D Is Upregulated During the Angiogenesis Following MI

In recent years, the histone methylation modification has been increasingly concerned in vascular biology [17]. However, as one of the many “readers” of the histone post-translational modifications (PTMs), histone methyltransferase KMT2D is rarely reported in cardiac angiogenesis. We first described protein expression in the myocardium tissue at different time points post-MI in mice and found that KMT2D expression was also upregulated in the first 7 days after MI and gradually decreased thereafter (Figs. 1D and E), which is consistent with the content of VEGF-A in myocardium, the abundance of CD31-positive endothelial capillaries, and α-SMA-positive arterioles. Meanwhile, the immunofluorescence staining results also showed that the positive signal of KMT2D protein expression in the LV section of mice increased first and then decreased in the post-MI myocardial remodeling process (Figure S2A). Despite the complexity of the regional myocardial tissue, both cardiomyocytes and ECs are essential for proper cardiac function and angiogenesis. As a consequence, we attempted to summarize the roles of KMT2D in angiogenesis focusing on cardiomyocytes and ECs following MI. To investigate which cell population is actually expressing KMT2D, the hypoxia models of cardiomyocytes and ECs were constructed to simulate ischemia in vitro, and the KMT2D protein expression at different time points of hypoxia were detected by Western blotting. HIF-1α was immediately induced in H9c2 cardiomyocytes at 1% oxygen concentration, followed by upregulation of VEGF-A expression, which is attributed to the transcriptional activation by HIF-1α (Figs. 1F and G). It is noteworthy that KMT2D was transiently up-regulated in early periods (hypoxia 6 h) but returned to the baseline after 18 h. On the other hand, the expression of KMT2D in ECs showed similar dynamic changes during hypoxia stimulation (Figs. 1H and I). Based on the above results, we propose that KMT2D may be involved in the protection cardiomyocytes and ECs under myocardial infarction and hypoxia and play a crucial role in the angiogenesis following MI.

### KM2TD Deficiency Aggravates Cardiac Dysfunction in MI Mice

**Fig. 2:**
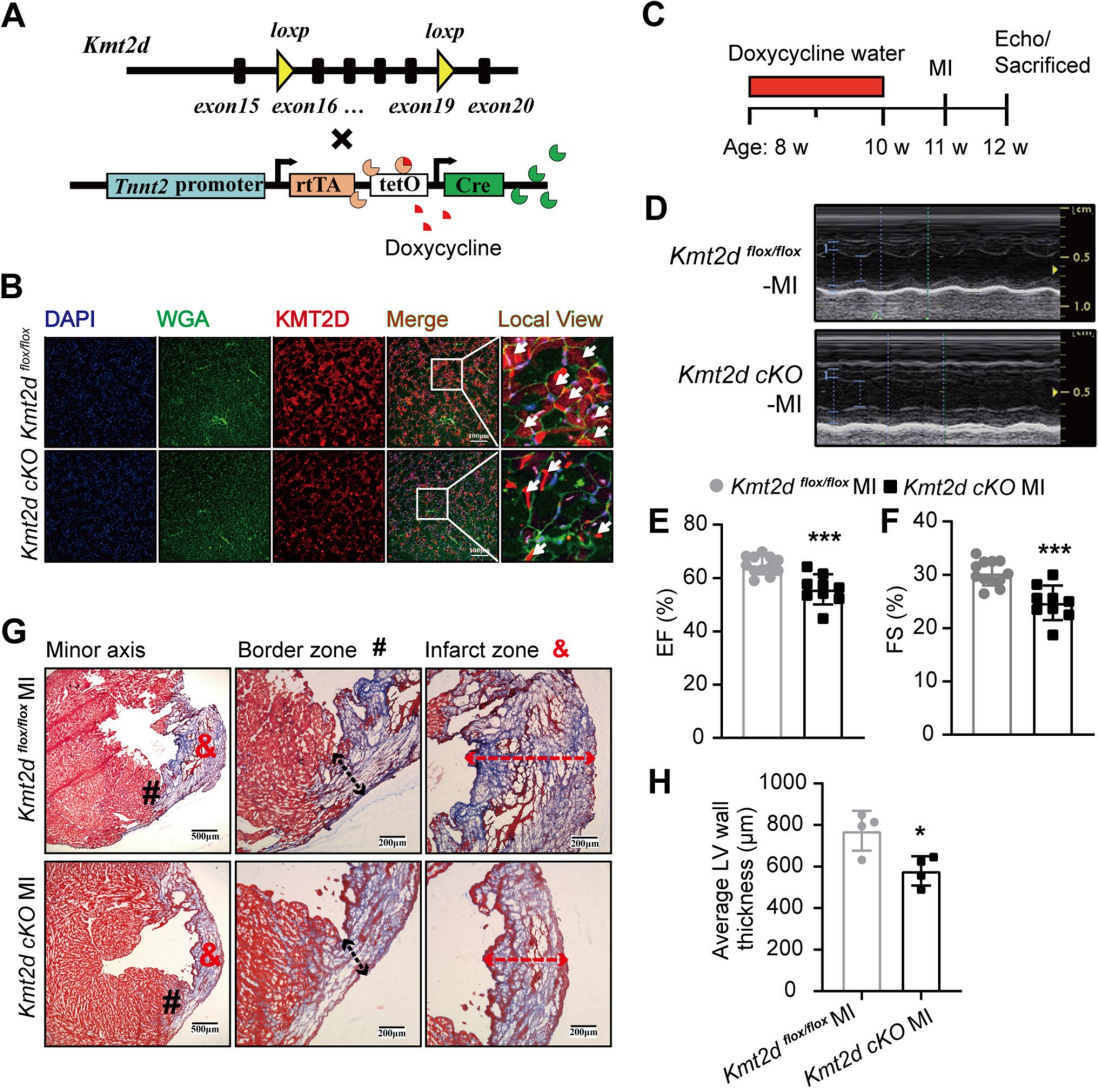
KM2TD deficiency aggravates cardiac dysfunction in MI mice. **A** Breeding scheme for cardiomyocyte-specific KMT2D knockout mice (*Kmt2d *^*flox/flox*^; *Tnnt2*-Cre): *Kmt2d *^*flox/flox*^ mice (top), *Tnnt2-rtTA;TetO-Cre* transgenic mice (bottom). **B** Representative immunofluorescent imaging of myocardial tissue sections from *Kmt2d*-cKO and littermates *Kmt2d *^*flox/flox*^ control mice (white arrow: KMT2D-positive cells; scale bar = 100 μm). **C** Timeline of doxycycline dosing, MI injury, and experimental endpoints. **D** Representative M-mode echocardiograms. **E**, **F** EF% and FS% from different groups of mice (*n* = 12: 9). **G** Representative images of Masson’s trichrome stained from *Kmt2d*-cKO and littermates *Kmt2d *^*flox/flox*^ control mice myocardial tissue sections (*n* = 4, scale bar = 500 μm and 200 μm; #: border zone, &: infarct zone). **H** Quantification of average LV wall thickness (*n* = 4). Data are shown as mean ± SD. Independent-sample *t* test (two-tailed) was used for statistical comparisons between 2 groups. ^*^P < 0.05

To determine the role of KMT2D in regulating MI-induced angiogenesis, we crossed *Kmt2d *^*flox/flox*^ mice with *Tnnt2-rtTA;TetO-Cre* transgenic mice to generate *Kmt2d *^*flox/flox*^; *Tnnt2*-Cre mice (Fig. 2A). The deletion efficiency of KMT2D in the heart tissue was detected 1 week after doxycycline feeding in the 11-week-old *Kmt2d *^*flox/flox*^; *Tnnt2*-Cre (*Kmt2d-*cKO) mice. Western blotting from LV tissue lysate showed the robust reduction of KMT2D in *Kmt2d-*cKO mice (Figure S2B). Magnified images of heart sections stained with WGA and KMT2D also showed that the positive signals of KMT2D in the *Kmt2d-*cKO mouse myocardium were significantly reduced, and most of the existing positive signals were colocalized with non-cardiomyocytes (Fig. 2B). Moreover, *Kmt2d-*cKO mice showed a normal cardiac function and heart morphology under basal condition (Figure S2D-G). To determine the role of KMT2D in pathological cardiac remodeling process following MI, we performed the following experiments. After 3 weeks of doxycycline induction, MI was induced in *Kmt2d-*cKO and littermates *Kmt2d *^*flox/flox*^ mice by the ligation of LAD. One week later, cardiac function in all mice were evaluated by echocardiography before the animals were sacrificed (Fig. 2C). Cardiac echocardiography demonstrated that *Kmt2d-*cKO mice exhibit an aggravated LV dysfunction, as manifested by significant reductions in EF% and FS% (Figs. 2D–F). Moreover, it appeared to be a slight expansion in the LV internal diameter (LV internal diastolic/systolic diameter, LVIDd/LVIDs) and a slight increase in end-diastolic volume (EDV) and end-systolic volume (ESV) of *Kmt2d-*cKO mice compared with the *Kmt2d *^*flox/flox*^ mice (Figure S2H, I). Consistently, histopathological analysis with Masson’s trichrome staining and quantification of the average wall thickness of the LV infarct zone (red dotted arrow) and the border zone (black dotted arrow), which was significantly thinner in the *Kmt2d-*cKO mice than the littermates *Kmt2d *^*flox/flox*^ mice (Figs. 2G and H).

### KM2TD Deficiency Accelerates Apoptosis of Myocardia and Attenuates Angiogenesis in MI Mice

**Fig. 3:**
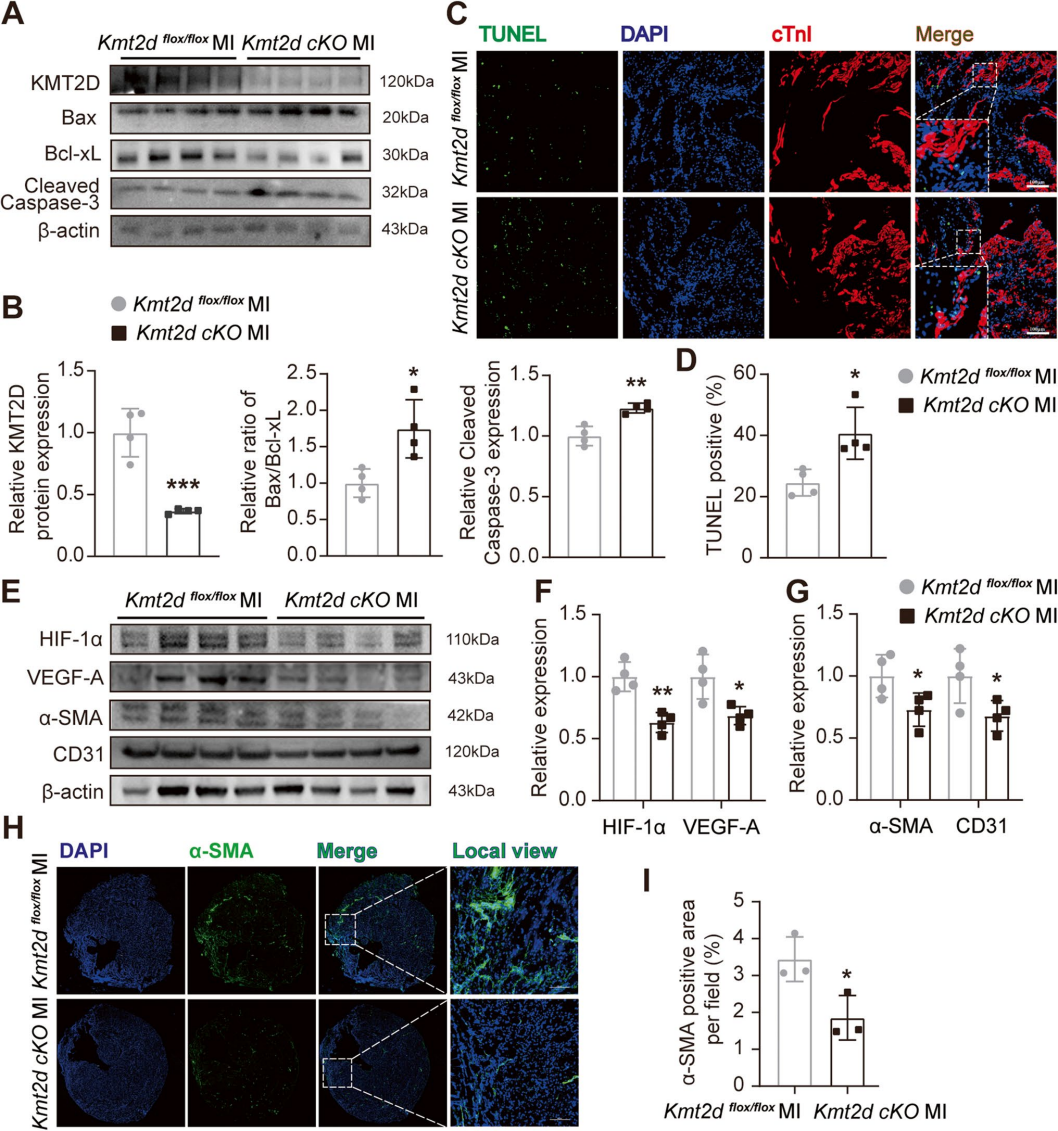
Cardiomyocyte-specific loss of KMT2D attenuates angiogenesis and accelerates cardiomyocyte apoptosis. **A** Representative images of Western blotting and **B** quantification analysis of apoptosis-related proteins expression in myocardial tissue of *Kmt2d*-cKO and littermates *Kmt2d *^*flox/flox*^ control mice 7 days after MI (*n* = 4). **C** Representative images of immunofluorescent staining for TUNEL and cTnI in myocardial tissue from *Kmt2d*-cKO and littermates *Kmt2d *^*flox/flox*^ control MI mice (*n* = 4, scale bar = 100 μm). **D** Quantification of TUNEL-positive cell rate (%). **E** Representative images of Western blotting and **F** quantification analysis of angiogenesis-related signaling protein expression in myocardial tissue of *Kmt2d*-cKO and littermates *Kmt2d *^*flox/flox*^ control mice 7 days after MI (*n* = 4). **G** Representative images of α-SMA immunofluorescent staining of myocardial tissue sections from *Kmt2d*-cKO and littermates *Kmt2d *^*flox/flox*^ control MI mice (*n* = 3, scale bar = 100 μm). **H** α-SMA positive area ratio based on immunofluorescent staining. Data are shown as mean ± SD. Independent-sample *t* test (two-tailed) was used for statistical comparisons between 2 groups. ^*^P < 0.05 and.^**^P < 0.01

The degree of myocardial apoptosis is negatively correlated to the thickness of the ventricular wall and to cardiac contractility, suggesting that ventricular dysfunction is attributed to the myocardial apoptosis [18, 19]. To investigate whether the KMT2D deficiency alters myocardial apoptosis after MI, we determined the expression of different anti- and pro-apoptotic markers in the border zone of LV using Western blotting. As shown in Fig. 3A, the protein expression levels of Bax and cleaved caspase-3 were upregulated, and the Bcl-xL was decreased in the *Kmt2d-*cKO mice as expected (Figs. 3A and B). TUNEL staining also revealed that the apoptosis rate of myocardial cells was significantly higher in the *Kmt2d-*cKO MI group than that in the *Kmt2d *^*flox/flox*^ MI group in the border zone (Fig. 3C and D). As is well known, early angiogenesis in ischemic myocardium can establish an effective collateral circulation to protect against myocardial cell death and myocardial remodeling after MI [20, 21]. Based on the consensus as well as our research results, we hypothesized that insufficient angiogenesis or inadequate formation of collateral circulation following MI seems to be a cause for excessive myocardial apoptosis in *Kmt2d-*cKO mice, at least partially. To further prove this hypothesis, the protein expression of HIF-1α/VEGF-A signaling pathway and capillaries and arteriole markers in myocardial tissue were detected. As shown in Fig. 3E, HIF-1α/VEGF-A signaling pathway-related proteins were downregulated in the *Kmt2d-*cKO mice. Furthermore, α-SMA and CD31 protein content was significantly lower in the *Kmt2d-*cKO mice than that in their littermates (Figs. 3E and G). More intuitive immunofluorescence staining results of myocardial tissue sections also proved that the α-SMA-positive vascular density in the LV of *Kmt2d-*cKO mice was relatively lower than that in the littermates (Figs. 3H and I). These data demonstrated that myocardial KMT2D deficiency attenuated angiogenesis and collateral development in mice subjected to MI and led to excessive myocardial apoptosis and further deterioration of cardiac function.

### Conditioned Medium from KMT2D-Deficient Hypoxic Cardiomyocytes Attenuates Endothelial Function

**Fig. 4:**
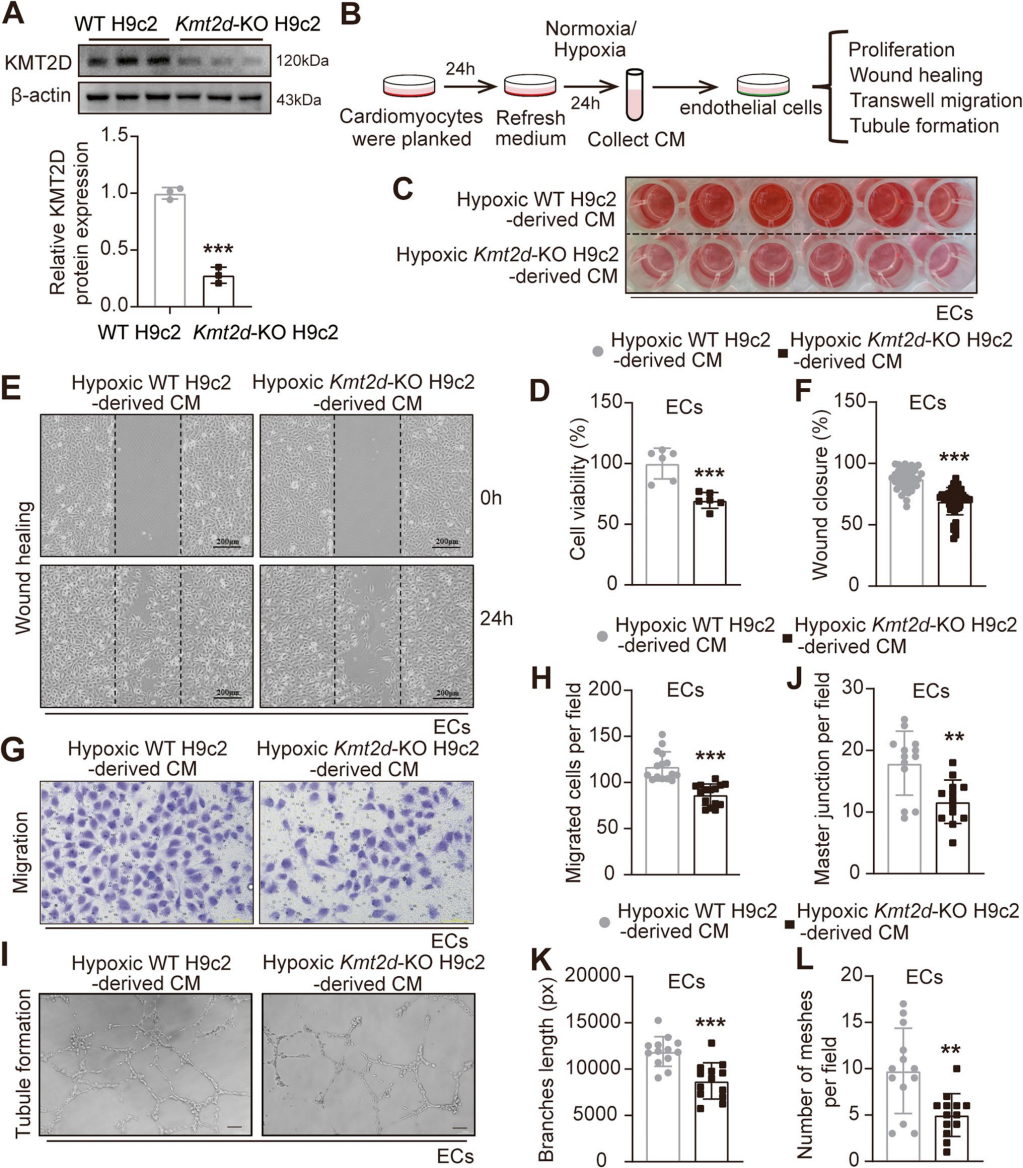
Conditioned medium from KMT2D-deficient cardiomyocytes impairs endothelial function. **A** Representative western blotting images of KMT2D expression in WT H9c2 and CRISPR/Cas9 gene-edited *Kmt2d*-KO H9c2 cardiomyocytes, with quantification of KMT2D protein expression (*n* = 3). **B** Experimental setup of cardiomyocyte paracrine effect on ECs. **C** CCK8 assay was used to evaluate the viability of ECs incubated with CM of WT H9c2 and *Kmt2d*-KO H9c2 cardiomyocytes for 24 h (*n* = 6), and **D** the statistical analysis of ECs viability (%). **E** Representative wound healing assay image of ECs incubated with CM of WT H9c2 and *Kmt2d*-KO H9c2 cardiomyocytes at 0 and 24 h, and **F** rate of wound area closure after 24 h (%) (*n* = 3, scale bar = 200 μm). **G** Representative migration images of ECs incubated with CM of WT H9c2 and *Kmt2d*-KO H9c2 cardiomyocytes for 48 h (*n* = 3, scale bar = 100 μm), and **H** quantitative analysis of the number of migrated cells. **I** Representative tube formation images of ECs incubated with CM of WT H9c2 and *Kmt2d*-KO H9c2 cardiomyocytes for 8 h (*n* = 3, scale bar = 100 μm), and **J** quantitative analysis of master junction, **K** branch length, and **L** number of meshes per field. Data are shown as mean ± SD. Independent-sample *t* test (two-tailed) was used for statistical comparisons between 2 groups. ^*^P < 0.05, ^**^P < 0.01, and.^***^P < 0.001

The intercellular communication between ECs and cardiomyocytes is critical for angiogenesis after MI [15]. Suppressed vascular density of *Kmt2d* cardiomyocyte-specific knockout mice after MI may be attributed to the inhibition of the paracrine effects of cardiomyocytes on ECs mediated by KMT2D deficiency. To this end, *Kmt2d* gene was deleted in H9c2 cardiomyocytes using the CRISPR/Cas9 gene-editing knockout system as previously reported [14], after which the CM was collected from the cardiomyocytes cultured for 24 h under normoxia or hypoxia (1% O_2_) and added into EC culture. After determining that KMT2D protein was significantly knocked down in H9c2 cells (Fig. 4A), cross-talk experiments were conducted according to the experimental setup to investigate whether the loss of KMT2D could affect the paracrine effects of cardiomyocytes on ECs (Fig. 4B). CCK-8 assay showed a significant decrease in ECs viability when treated with CM from hypoxic *Kmt2d-*KO cardiomyocytes (Figs. 4C and D). We then analyzed EC migration using wound healing and Transwell assay. We found that hypoxic *Kmt2d-*KO cardiomyocytes-CM was associated with the attenuated wound areas and migration of ECs across the membrane compared with that of WT cardiomyocytes-CM (Figs. 4E–H). Tubule formation was assessed in ECs exposed to cardiomyocytes-CM (Fig. 4I). As shown in Figs. 4J–L, the statistical graph of capillary network showed that the number of master junction, the length of branches, and the number of meshes in the *Kmt2d-*KO cardiomyocytes-CM cultured ECs were significantly reduced compared to the WT cardiomyocytes-CM. In addition, ECs incubated with *Kmt2d*-KO cardiomyocyte-derived normoxia CM also showed a poorer proliferation and wound healing ability compared to the WT cardiomyocytes-CM, while there was no difference in tubule formation and migration (Figure S3E-J). These findings indicate that KMT2D silencing in cardiomyocytes impaired the normal EC function and reduced the endothelial angiogenesis capability at least partially via a paracrine pathway.

### KM2TD Deficiency Suppresses the Transcriptional Activation of Vegf-a in Cardiomyocytes

**Fig. 5:**
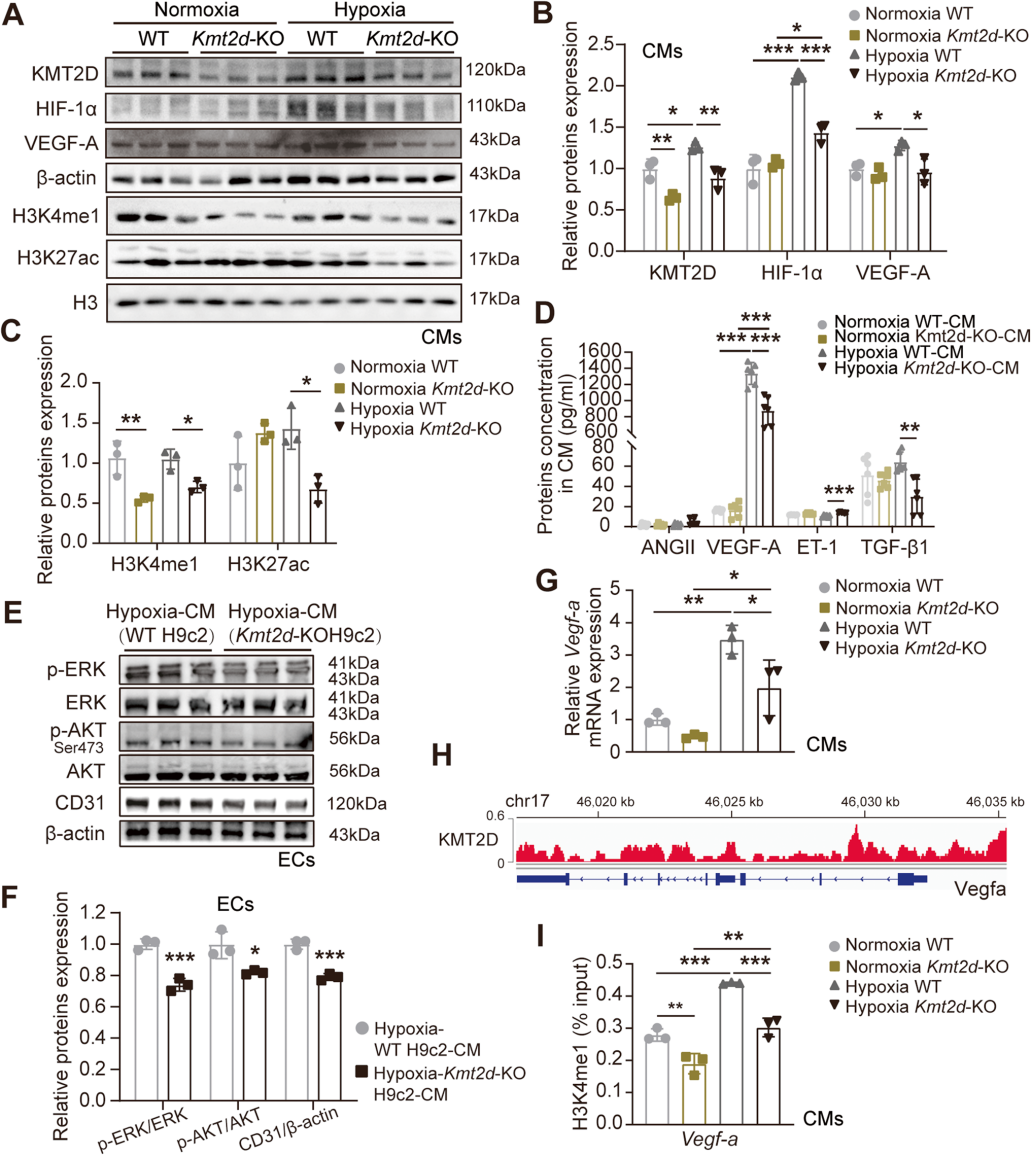
KMT2D silencing suppresses the transcriptional activation of *Vegf-a* in cardiomyocytes. **A** Representative images of western blotting and **B**, **C** quantification analysis of protein expression in WT H9c2 and *Kmt2d*-KO H9c2 cardiomyocytes under normoxia and hypoxia treatment (*n* = 3). **D**Quantitative analysis of the concentrations of angiogenic growth factors in CM of WT H9c2 and *Kmt2d*-KO H9c2 cardiomyocytes under normoxia and hypoxia treatment (*n* = 3). **E** Representative images of western blotting and **F** quantification analysis of ERK and AKT signaling pathways protein expression in ECs incubated with hypoxic WT or *Kmt2d*-KO H9c2 cardiomyocytes-derived CM (*n* = 3). **G** RT-qPCR analysis for *Vegf-a* mRNA in H9c2 and *Kmt2d*-KO H9c2 cardiomyocytes under normoxia and hypoxia treatment (*n* = 3). **H** IGV tracks revealing the results of CUT-Tag reads (KMT2D binding) distributions in *Vegfa* of the WT mouse heart sample. **I** CUT&Tag-qPCR validation of the distribution of H3K4me1 changes in *Vegf-a* promoter (*n* = 3). Data are shown as mean ± SD. Independent-sample *t* test (two-tailed) was used for statistical comparisons between 2 groups. One-way ANOVA followed by Tukey’s post hoc test was used for statistical comparisons between multiple groups. ^*^P < 0.05, ^**^P < 0.01, and.^***^P < 0.001

In order to further explore whether the KMT2D deletion suppressed the transcriptional activation of genes that control angiogenesis and ultimately blocks the transmission of proangiogenic signals secreted in cardiomyocytes, we analyzed KMT2D protein expression under both normoxia and hypoxia conditions and observed a significant reduction in the *Kmt2d-*KO cardiomyocytes (Fig. 5A and B), accompanied with significantly reduced expression of HIF1α/VEGF-A signaling pathway-related proteins, compared to the WT cardiomyocytes (Figs. 5A and B). Further analyses of the content of growth factors in the supernatant of CM from WT cardiomyocytes revealed that VEGF-A was significantly induced and secreted into the supernatant with hypoxia (Fig. 5D), whereas KMT2D deficiency resisted hypoxia-induced VEGF-A secretion. Coincidentally, the TGF-β1, which also drives endothelial proliferation, migration, and stimulates angiogenesis, was also significantly reduced due to KMT2D deletion. Ang II was used as a negative control, and no effective content was detected. In addition, ET-1 was upregulated in the supernatant of KMT2D knockout cardiomyocytes, suggesting that KMT2D deletion may cause cardiomyocytes injury (Fig. 5D). We further examined other proteins that participate in the protection against MI injury and EC function [22–25] and found that the active phosphorylated ERK (p-ERK) and phosphorylated AKT (p-AKT) levels of ECs cultured in hypoxic CM from *Kmt2d-*KO cardiomyocytes were lower than that of WT cardiomyocytes-CM and that the mature EC marker CD31 was also lower than WT cardiomyocytes-CM group (Figs. 5E and F). These data confirm that KMT2D deletion suppressed the expression and secretion of some growth factors in cardiomyocytes, ultimately inhibited the paracrine effects of the cardiomyocytes and affected neighboring ECs.

Monomethylation of histone H3 at lysine 4 (H3K4me1) and acetylation of histone H3 at lysine 27 (H3K27ac) are generally considered the hallmark of genes transcriptional activation [26]. Our results suggest that loss of KMT2D was associated with the decrease in global H3K4me1and H3K27ac marker in hypoxic cardiomyocytes (Fig. 5A and C). Because the RT-qPCR results showed that hypoxia induced up-regulation of *Vegf-a* mRNA level, whereas *Vegf-a* mRNA level were reversed in the *Kmt2d-*KO cardiomyocytes (Fig. 5G), we hypothesized that the expression of *Vgef-a* is either directly or indirectly regulated by the KMT2D. The results of CUT&Tag reads distribution of KMT2D occupancy peaks was shown by Integrative genomics viewer (IGV) on *Vegf-a* transcriptional start site (TSS) region and promoter region (located between about 46,030–46,035 kb) (Fig. 5H). Moreover, as shown in Fig. 5I, H3K4me1 CUT&Tag-qPCR results indicated that hypoxia increased the H3K4me1 binding on *Vegf-a* promoter. However, KMT2D silencing decreased the H3K4me1 binding. To sum up, the results indicated that KMT2D deletion blocked *Vegf-a* transcription by reducing H3K4me1 level bound on *Vegf-a* promoter in cardiomyocytes.

In order to exclude the decreased binding of HIF-1α to the hypoxia-responsive element (HRE) on the *Vegf-a* gene and thus affects the expression of *Vegf-a* mRNA, we knocked out the binding site (-TACGTG-) in the *Vegf-a* promoter region of H9c2 cardiomyocytes by CRISPR/Cas9 gene-editing knockout system and detected the expression level of *Vegf-a* mRNA under hypoxia. As shown in Figure S4A, the absence of binding site suppresses hypoxia-induced *Vegf-a* mRNA expression, but hypoxia still induces the significant up-regulation of *Vegf-a* mRNA compared with normoxic H9c2. Moreover, the increased expression of *Kmt2d* in HIF-1α binding site knocked out H9c2 is observed, suggesting that more KMT2D may be required to promote *Vegf-a* transcription to rescue the deficiency of *Vegfa* (Figure S4A).

### KMT2D Is Essential for the Maintenance of Normal Endothelial Function Through Regulating VEGF-A Transcription in Endothelial Cells

**Fig. 6:**
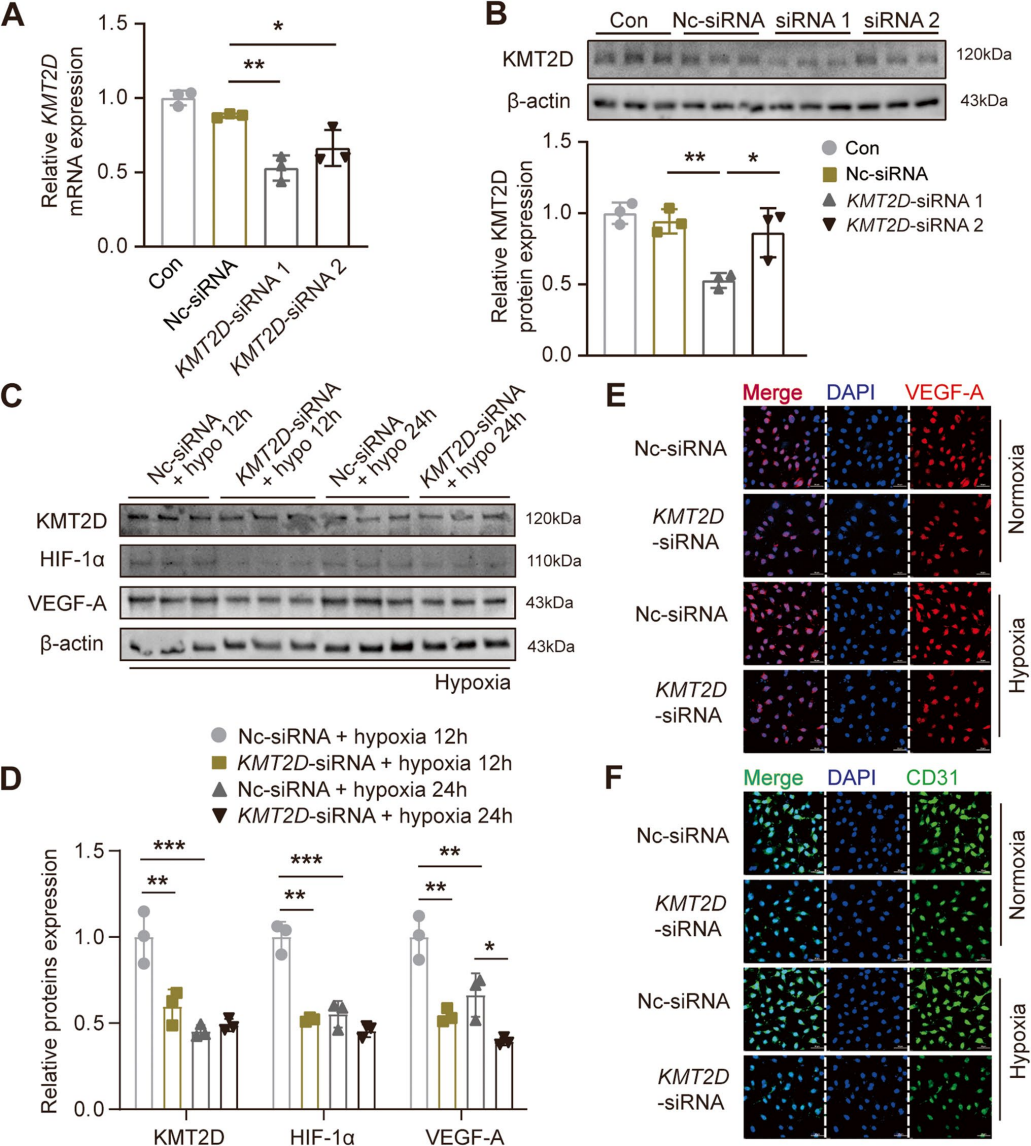
KMT2D deficiency in endothelial cells inhibits HIF-1α/VEGF-A signaling pathway. **A** RT-qPCR analysis for *KMT2D* mRNA in EA.hy926 ECs to verify knockdown efficiency of siRNA (*n* = 3). **B** Representative western blotting images of KMT2D expression in EA.hy926 ECs with quantification of KMT2D protein expression (*n* = 3). **C** Representative images of western blotting and **D** quantification analysis of HIF-1α/VEGF-A signaling pathway proteins expression in ECs with hypoxia treatment (*n* = 3). **E** Representative images of VEGF-A immunofluorescent staining (represented by red fluorescent signals) of ECs under normoxia and hypoxia conditions (scale bar = 50 μm). **F** Representative images of CD31 immunofluorescent staining (green fluorescent signals) of ECs under normoxia and hypoxia conditions (scale bar = 50 μm). Data are shown as mean ± SD. One-way ANOVA followed by Tukey post hoc test was used for statistical comparisons between multiple groups. ^*^P < 0.05, ^**^P < 0.01, and.^***^P < 0.001

Considering that the protein expression of KMT2D in ECs is also sensitive to persistent hypoxia (Figs. 1H and I), we further clarified the direct role of KMT2D in ECs. Firstly, ECs were transfected with siRNA duplexes corresponding to *KMT2D* to knockdown its expression. As shown in Figs. 6A and B, the introduction of *KMT2D*-siRNA-1 effectively inhibited the mRNA expression and significantly reduced the expression of KMT2D protein. The results in Fig. 1I show that the protein expression of KMT2D peaked at about 6–12 h after hypoxia treatment and then returned to basal level by 24 h. As a consequence, we further explored the function of KMT2D in ECs at two time points (12 h and 24 h) of hypoxia. Statistical analysis of the Western blotting results revealed that after 12-h hypoxia treatment, HIF-1α/VEGF-A signaling pathway proteins were inhibited due to KMT2D silencing. Importantly, more prolonged hypoxia (24 h) reduced the protein expression of KMT2D, HIF-1α, and VEGF-A, while KMT2D silencing further inhibited the VEGF-A protein expression (Fig. 6C and D). In addition, KMT2D silencing can play the same role in normoxia condition (Figure S4B, C), with that in the hypoxia. Immunofluorescence staining further confirmed that KMT2D silencing reduced the fluorescence intensity of VEGF-A and CD31-positive signals under both normoxia and hypoxia (Figs. 6E and F and Figure S4E, F).

**Fig. 7:**
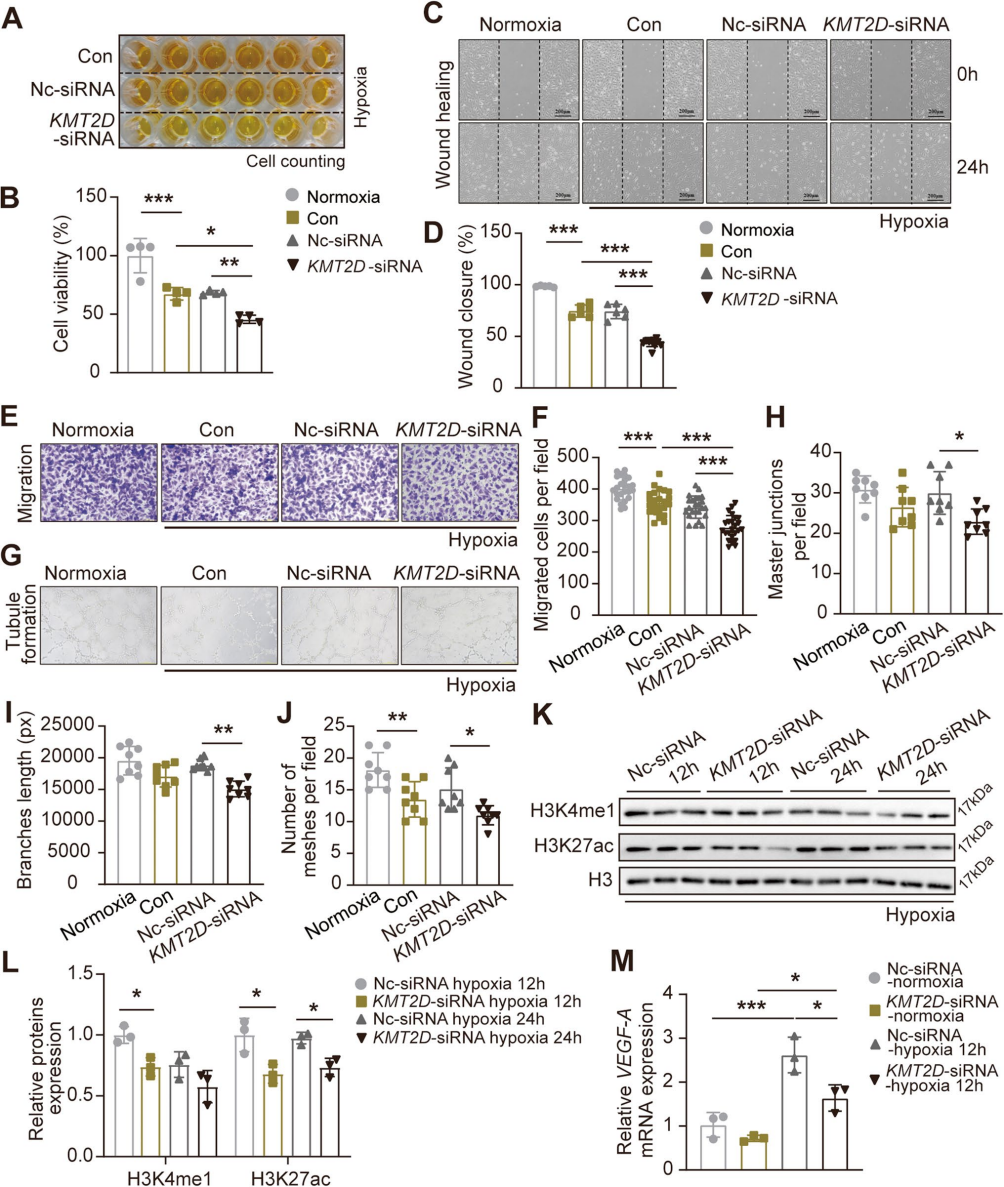
KMT2D is essential for the maintenance of normal endothelial function and *VEGF-A* transcription in endothelial cells. **A** CCK8 assay was used to evaluate the viability of *KMT2D* silenced and control ECs (*n* = 6), and **B** the statistical analysis of ECs viability (%). **C** Representative wound healing assay image of *KMT2D* silenced and control ECs at 0 and 24 h and **D** rate of wound area closure after 24 h (%) (*n* = 3, scale bar = 200 μm). **E** Representative migration images of *KMT2D* silenced and control ECs for 24 h (*n* = 3, scale bar = 100 μm), and **F** quantitative analysis of the number of migrated cells. **G** Representative tube formation images of *KMT2D* silenced and control ECs (*n* = 3, scale bar = 100 μm), and quantitative analysis of **H** master junction, **I** branch length, and **J** number of meshes per field. **K** Representative images of Western blotting and **L** quantification analysis of global H3K4me1 and H3K27ac protein expression in ECs under hypoxia treatment (*n* = 3). **M** RT-qPCR analysis for *VEGF-A* mRNA in ECs (*n* = 3). Data are shown as mean ± SD. One-way ANOVA followed by Tukey’s post hoc test was used for statistical comparisons between multiple groups. ^*^P < 0.05, ^**^P < 0.01, and.^***^P < 0.001

To investigate whether KMT2D in ECs affects angiogenesis, we evaluated the function of KMT2D-silenced endothelial cells. As shown in Figs. 7A and B, reduced expression of KMT2D by *KMT2D*-siRNA transfection further suppressed EC proliferation by 22.8% compared with Nc-siRNA control, which is basically consistent with the results under normoxia condition (Figure S5A, B). In addition, KMT2D silencing also inhibited EC migration compared with Nc-siRNA under both normoxia and hypoxia conditions, which were demonstrated by wound healing (Figure S5C, D and Figs. 7C and D) and Transwell migration assay (Figure S5E, F and Figs. 7E and F). The Matrigel-based tubule formation assay showed that *KMT2D*-siRNA transfection significantly inhibited tube formation in both normoxia (Figure S5G-J) and hypoxia (Figs. 7G–J), with less numbers of master junction, shorter length of branches, and less numbers of meshes. Collectively, these data suggest that KMT2D played an important role in regulating the HIF-1α/VEGF-A signaling pathway which promotes proliferation, migration, and angiogenesis of ECs. In order to investigate the molecular mechanism of KMT2D involved in the regulation of ECs function, we also identified the significant reduction of global histone H3 monomethylation and acetylation levels in ECs after KMT2D silencing under normoxia (Figure S4B, D) and hypoxia (Figs. 7 K and L). These results were similar with that in the cardiomyocytes (Figs. 5A and C). RT-qPCR results also indicated that the mRNA level of *VEGF-A* was significantly reduced after KMT2D was silenced (Fig. 7 M), suggesting that the deletion of KMT2D in ECs resulted in the inhibition of *VEGF-A* transcription, and in other words, KMT2D is essential for *VEGF-A* transcription and the maintenance of normal endothelial function in ECs.

## Discussion

Heart disease remains the leading cause of death worldwide. Discovery of new key regulatory molecules participating in angiogenesis after MI could facilitate to the development of better therapeutic interventions. As a key regulatory mechanism of gene transcription, histone methylation modification plays a critical role in the occurrence and development of heart diseases.^8^ Yang et al. reported that SUV39H regulates ROS levels in the MI heart in a SIRT1-dependent manner, and SUV39H gene silencing protects against myocardial infarction in mice [27]. Furthermore, histone methyltransferase G9a has been reported to suppress the transcription of antihypertrophic genes by regulating the trimethylation level of H3K27 [28].

As an important survival mechanism, the formation of capillaries and coronary collateral vessels is adaptive angiogenic response in the heart after MI. However, there are few reports on angiogenesis after MI from the perspective of histone methylation modifications [29]. A study using zebrafish as a model demonstrates that KMT2D-Notch signaling pathway is involved in the regulation of endocardium vasculogenesis and angiogenesis [30]. Increasing evidence showed that histone methylation modification and its responsible methyltransferases or demethylases are crucial in the endothelial cell function and angiogenesis process. For instance, Song et al. have demonstrated that NSD2, a histone methyltransferase, can enhance tumor angiogenesis via the activation of STAT3 [31]. Meanwhile, the histone demethylase PHF8 regulates the survival, proliferation, and migration of ECs by regulating the expression of E2F4 [32]. Furthermore, histone methyltransferase SET7 interacts with GATA-1 to promote VEGF-A transcription and angiogenesis [33]. However, whether histone methylation modifications and their corresponding HMTs or KDMs are involved in the regulation of angiogenesis in MI heart needs to be further explored.

KMT2D is involved in regulating chromatin accessibility and ultimately regulating gene transcriptional activation by maintaining global H3K4me1 level [34]. Loss of KMT2D protein significantly reduces the transcription activity of target genes [35]. Our previous studies demonstrated that cardiomyocyte-specific KMT2D knockout mice exhibited larger infarct size after myocardial infarction surgery with reduced expression of functional genes [14]. In the current study, we detected impaired angiogenesis and reduced level of H3K4me1 in the *Vegf-a* promoter region during MI in the cardiomyocyte-specific *Kmt2d* knockout mice and KMT2D silenced H9c2 cardiomyocyte respectively. This phenomenon was also observed in ECs by silencing the expression of *Kmt2d* gene. These data indicates that KMT2D-mediated histone methylation modifications in cardiomyocytes could contribute to the transcriptional activation of *Vegf-a* and angiogenesis, and KMT2D is also required for the maintenance of normal endothelial function.

*Kmt2d* complete knockout mice showed early embryonic lethality [36]. Using *Kmt2d-*cKO mice, we further clarified that KMT2D is indispensable in the heart, because the loss of KMT2D exacerbated heart failure and increases the rate of cardiomyocytes apoptosis after MI. Moreover, excessive cardiomyocyte apoptosis is also associated with insufficient angiogenesis after MI [20]. At the same time, KMT2D may have a direct effect on cardiomyocyte viability itself, and we will conduct further research in the future.

It has been considered that there is an extensive intercellular communication network between cardiomyocytes and non-cardiomyocytes, and the cellular cross-talk is essential for cardiac repair and angiogenesis after MI.^15^ Some secreted proteins from the heart are required to maintain normal cardiac function and regulate ventricular remodeling after myocardial injury. The communication paths between cardiomyocytes and ECs during myocardial injury is mainly manifested in the secretion of proangiogenic factors to promote adaptive vascular growth [15, 37]. In the in vitro setting, collecting conditioned medium obtained from cardiomyocytes provides a useful means to study the paracrine mechanisms of cardiomyocytes on other cells after performing co-cultures [38]. Shi et al. reported that early hypoxic cardiomyocyte metabolites TNF-α and IL-1β can induce the migration of cardiac fibroblasts [39]. Xie et al. found that cardiomyocyte medium for hypoxic preconditioning can induce MSC differentiation into myocardial-like cells [40]. Moreover, it has been reported that hypoxia facilitated the production of transforming growth factor-beta (TGF-β) in H9c2 cell-derived exosomes [41]. The above reports suggest that there are factors and exosome that are released from the cardiomyocytes subjected to hypoxia that has an important cardioprotective role in limiting myocardial damage. Our data demonstrated that KMT2D-deficient cardiomyocyte-derived CM suppressed EC function accompanying with reduced H3K4me1 level and VEGF transcription in the cardiomyocytes response to MI or hypoxia, suggesting the close intercellular communication between cardiomyocytes and ECs.

Activation of endothelial PI3K/AKT and MEK/ERK pathways is essential in VEGF-A-induced angiogenesis by regulating cell survival and migration [42, 43]. The impaired endothelial function we observed was corresponded to the reduced p-AKT and p-ERK protein levels in the ECs cultured with KMT2D-deficient cardiomyocytes-derived CM. These results reveal that KMT2D, as a crucial cardioprotective factor, affects not only myocardium but also the surrounding ECs during MI and provide insight into the paracrine signaling between cardiomyocytes and ECs.

In this study, we also evaluated the direct effect of KMT2D on endothelial function. Similar with cardiomyocytes, the decreased expression of HIF-1α/VEGF-A signaling pathway proteins in ECs due to KMT2D silencing was also observed. Our results also found that KMT2D silencing inhibited ECs proliferation, migration, and tubule formation function under hypoxia, which may be attributed to the direct inhibition of VEGF-A expression after KMT2D silencing. However, in contrast to the in vivo results, we did not observe that hypoxia induced increased vasculogenic ability of ECs, which may be related to the fact that we did not use primary vascular endothelial cells and the hypoxic treatment time in our experiments. Finally, we concluded that KMT2D regulates H3K4me1 modification in ECs through methyltransferase function and regulates EC function. To verify this finding, it might be necessary to generate cardiac microvascular endothelial cells cardiac microvascular endothelial-specific *Kmt2d* knockout mice and explore the specific regulatory mechanism of KMT2D in endothelial cells in vivo.

The optimal observation time for angiogenesis in mouse MI model is controversial. Karina et al. proposed that the capillary network was significantly dilated at day 7 post-MI [44]. Fan et al. evaluated myocardial tissue angiogenesis 4 weeks after MI [45]. In this study, we evaluated capillaries and arterioles densities in myocardial tissue of MI mice at different time points by immunofluorescence staining, and concluded that day 3 to day 7 post-MI is the critical stage of neovascularization accumulation (Figure S1C-F). This is consistent with the previously observed pattern of endothelial activation and dilated capillaries in the ischemic myocardial region.^44^ At the same time, we also observed that the expression level of KMT2D in myocardial tissue, cardiomyocytes, and ECs had a similar dynamic pattern of angiogenesis. These results provide strong support for us to explore the function of KMT2D in angiogenesis after MI.

In summary, our study indicates that KMT2D depletion in both cardiomyocytes and ECs attenuates angiogenesis and that loss of KMT2D in cardiomyocytes reduced their paracrine of VEGF-A and impaired the EC function through cross-talk. This study suggests that the strategy of targeting KMT2D could be a potential therapeutic approach for angiogenesis against myocardial ischemia and heart failure.

### Supplementary Information

Below is the link to the electronic supplementary material.

Supplementary file1 (DOCX 22 KB)

Supplementary file2 (DOCX 18 KB)
