# Supplementary material for: Loss of Histone Methyltransferase KMT2D Attenuates Angiogenesis in the Ischemic Heart by Inhibiting the Transcriptional Activation of VEGF-A
Source: J Cardiovasc Transl Res. 2023 Mar 22;16(5):1032–49. doi: 10.1007/s12265-023-10373-x (PMC10616223; doi:10.1007/s12265-023-10373-x)
Supplement: Supplementary file 1 — Supplementary file1 (DOCX 22 KB) [file 12265_2023_10373_MOESM1_ESM.docx]

**Supplementary Figure Legends**

**Supplementary Figure 1. Dynamic evaluation of angiogenesis after MI.** (A) The surgical ligation MI model is made by ligating the left anterior descending coronary artery (LAD). (B) Representative electrocardiogram (ECG) of sham and MI mice. (C) Representative images of cTnI and CD31 immunofluorescent staining in myocardial tissue sections of mice at different time points after MI (*n* = 6, scale bar = 100 μm). (D) Quantification analysis of relative CD31 positive area ratio based on immunofluorescent staining (*n* = 6, **P* < 0.05 vs sham; #*P* < 0.05, ##*P* < 0.01 vs MI 1d; &*P* < 0.05, &&&*P* < 0.001 vs MI 3d). (E) Representative images of cTnI and α-SMA immunofluorescent staining in myocardial tissue sections of mice at different time points after MI (*n* = 6, scale bar = 100 μm). (F) Quantification analysis of relative α-SMA positive area ratio based on immunofluorescent staining (*n* = 6, **P* < 0.05, ****P* < 0.001 vs sham; ##*P* < 0.01, ###*P* < 0.001 vs MI 3d; &&*P* < 0.01 vs MI 7d). (G) Representative images of cTnI and α-SMA immunofluorescent staining in myocardial tissue sections of mice at different time points after MI (scale bar = 100 μm); Data are shown as mean ± SD. One-way ANOVA followed by Tukey post hoc test was used for statistical comparisons between multiple groups.

**Supplementary Figure 2. Detection of knockout efficiency and cardiac function in *Kmt2d* myocardial specific knockout mice.** Representative images of KMT2D immunofluorescent staining in myocardial tissue sections of mice at different time points after MI (*n* = 6, scale bar = 100 μm). (B) Representative images of western blotting and (C) Quantification analysis of KMT2D proteins expression in WT and *Kmt2d*-cKO mice (*n* = 3). (D) Timeline of doxycycline dosing, Echocardiography and experimental endpoints. (E) Representative M-mode echocardiograms. (F) EF% and FS% from different groups of mice (*n* = 6 : 6). (G) Representative diagram of the general shape of the heart. (H) Left ventricular internal diastolic/systolic diameter (LVIDd, LVIDs) from different groups of mice (*n* = 12 : 9). (I) Left ventricular End-diastolic volume (EDV) and End-systolic volume (ESV) (*n* = 12 : 9). Data are shown as mean ± SD. Independent-sample t test (two-tailed) was used for statistical comparisons between 2 groups. ***P* < 0.01.

**Supplementary Figure 3. Conditioned medium derived from KMT2D-deficient cardiomyocytes** **under normoxia condition attenuated endothelial function.** (A) CCK8 assay was used to evaluate the viability of ECs incubated with conditioned medium of WT H9c2 and *Kmt2d*-KO H9c2 cardiomyocytes for 24 h (*n* = 6), and (B) the statistical analysis of ECs viability (%). (C) Representative wound healing assay image of ECs incubated with conditioned medium of WT H9c2 and *Kmt2d*-KO H9c2 cardiomyocytes at 0 and 24 h and (D) rate of wound area closure after 24 h (%) (*n* = 3, scale bar = 200 μm). (E) Representative migration images of ECs incubated with conditioned medium of WT H9c2 and *Kmt2d*-KO H9c2 cardiomyocytes for 48 h (*n* = 3, scale bar = 100 μm), and (F) quantitative analysis of the number of migrated cells. (G) Representative tube formation images of ECs incubated with conditioned medium of WT H9c2 and *Kmt2d*-KO H9c2 cardiomyocytes for 8 hours (*n* = 3, scale bar = 100 μm), and (H) quantitative analysis of master junction (I) branches length and (J) number of meshes per field. Data are shown as mean ± SD. Independent-sample t test (two-tailed) was used for statistical comparisons between 2 groups. **P*< 0.05; ****P* < 0.001.

**Supplementary Figure 4. KMT2D silencing in endothelial cells inhibits HIF-1α/VEGF-A signaling pathway.** (A) RT-qPCR analysis for *Vegfa* and *Kmt2d* mRNA in WT and *Vegfa* HRE (-TACGTG-) KO H9c2 (*n* = 4). (B) Representative images of western blotting and (C) quantification analysis of HIF-1α/VEGF-A signaling pathway proteins expression in ECs with normoxia treatment (*n* = 3). (D) Quantification analysis of global H3K4me1 and H3K27ac proteins expression in ECs under normoxia treatment (*n* = 3). (E) Quantification analysis of the fluorescence intensity of VEGF-A immunofluorescence staining. (F) Quantification analysis of the fluorescence intensity of CD31 immunofluorescence staining. Data are shown as mean ± SD. One-way ANOVA followed by Tukey post hoc test was used for statistical comparisons between multiple groups. **P* < 0.05; ***P* < 0.01; ****P* < 0.001.

**Supplementary Figure 5. KMT2D silencing in endothelial cells impairs endothelial function.** (A) CCK8 assay was used to evaluate the viability of *KMT2D* silenced and control ECs under normoxia condition (*n* = 6), and (B) the statistical analysis of ECs viability (%). (C) Representative wound healing assay image of *KMT2D* silenced and control ECs under normoxia condition at 0 and 24 h and (D) rate of wound area closure after 24 h (%) (*n* = 3, scale bar = 200 μm). (E) Representative migration images of *KMT2D* silenced and control ECs under normoxia condition (*n* = 3, scale bar = 100 μm), and (F) quantitative analysis of the number of migrated cells. (G) Representative tube formation images of *KMT2D* silenced and control ECs under normoxia condition (*n* = 3, scale bar = 100 μm), and (H) quantitative analysis of master junction, (I) branches length and (J) number of meshes per field. Data are shown as mean ± SD. One-way ANOVA followed by Tukey post hoc test was used for statistical comparisons between multiple groups. ***P* < 0.01; ****P* < 0.001.
