# Supplementary material for: Loss of Histone Methyltransferase KMT2D Attenuates Angiogenesis in the Ischemic Heart by Inhibiting the Transcriptional Activation of VEGF-A
Source: J Cardiovasc Transl Res. 2023 Mar 22;16(5):1032–49. doi: 10.1007/s12265-023-10373-x (PMC10616223; doi:10.1007/s12265-023-10373-x)
Supplement: Supplementary file 2 — Supplementary file2 (DOCX 18 KB) [file 12265_2023_10373_MOESM2_ESM.docx]

**Supplementary Table 1.** The sequences of human *KMT2D* siRNA.

|  | Sense 5'-3' | antisense 5'-3' |
| --- | --- | --- |
| *KMT2D-*siRNA-1 | GCAAAUCGCUAGCAUCAUUtt | AAUGAUGCUAGCGAUUUGCtt |
| *KMT2D-*siRNA-2 | GAGUCGAACUUUACUGUCUtt | AGACAGUAAAGUUGCACUCtt |

**Supplementary Table 2.** The primer sequences for qRT-PCR.

| Genes | Forward 5'-3' | Reverse 5'-3' |
| --- | --- | --- |
| H*-KMT2D*  R*-Kmt2d*  H*-VEGF-A*  R*-Vegf-a*  R*-Vegf-a* (Promoter) | CAGCTATCAGCGGAGGCAAA  GCGTTGTGTGGAGTGTATCG  ATCTTCAAGCCATCCTGTGTGC  ACTCATCAGCCAGGGAGTCT  TCTGTCGTACGTGGAGG | AGCAGTTGACTTTAGCCGGG  CACACCACTTGCACTTCCAG  GCTCACCGCCTCGGCTTGT  GGGAGTGAAGGAGCAACCC  GATCGTACGTGCGGTGACTC |
| H-*GAPDH*  R*-Gapdh* | TCGCCAGCCGAGCCA  ACAGCAACAGGGTGGTGGAC | GAGTTAAAAGCAGCCCTGGTG  TTTGAGGGTGCAGCGAACTT |

**Supplementary Table 3.** The antibody information for western blotting and immunofluorescent staining.

| Antibody | Cat No. | Producer | Dilution |
| --- | --- | --- | --- |
| Cardiac Troponin I ab56357 abcam 1:200 (IF) | | | |
| KMT2D ABE1867 Merck 1:1000/ 1:200 (IF) | | | |
| HIF-1α ab179483 abcam 1:1000 | | | |
| VEGF-A 66828-1 Proteintech 1:1000/ 1:200 (IF) | | | |
| H3K4me1 ab8895 abcam 1:1000 | | | |
| H3K27ac 8173s CST 1:1000 | | | |
| Bax 14796s CST 1:1000 | | | |
| Bcl-xL 2764s CST 1:1000 | | | |
| Cleaved Caspase-3 ET1608-64 HUABIO 1:1000 | | | |
| α-SMA GB111364 Servicebio 1:1000/ 1:200 (IF) | | | |
| CD31 GB11063-2 Servicebio 1:1000/ 1:200 (IF) | | | |
| ERK ET1601-29 HUABIO 1:1000 | | | |
| p-ERK ET1610-13 HUABIO 1:1000 | | | |
| AKT ET1609-51 HUABIO 1:1000 | | | |
| p-AKT ET1607-73 HUABIO 1:1000 | | | |
| H3 4499s CST 1:1000 | | | |
| β-actin 285336 Invitrogen 1:1000 | | | |
